# Intravitreal Anti–Vascular Endothelial Growth Factor Use in France During the Coronavirus Disease 2019 Pandemic

**DOI:** 10.1001/jamaophthalmol.2020.5594

**Published:** 2020-12-17

**Authors:** Sophie Billioti de Gage, Jérôme Drouin, David Desplas, François Cuenot, Rosemary Dray-Spira, Alain Weill, Mahmoud Zureik

**Affiliations:** 1 EPI-PHARE, French National Agency for Medicines and Health Products Safety, French National Health Insurance, Saint-Denis, France

## Abstract

This cohort study uses data from the French National Health Data System to assess the decline in the use of intravitreal anti–vascular endothelial growth factors before, during, and after pandemic-associated lockdown periods.

The coronavirus disease 2019 (COVID-19) pandemic emerged in France in February 2020. On March 17, 2020, a national population lockdown restricted human contacts and travel to a strict minimum until May 11, 2020. Many patients experienced difficulties or fear of accessing health care during lockdown.^1^ We hypothesized that the COVID-19 pandemic may have modified the dispensing of intravitreal (IVT) anti–vascular endothelial growth factors (anti-VEGF), the main treatment for retinal vascular abnormalities.^2^ This study quantified changes in the use of IVT anti-VEGF since the pandemic began in France.

## Methods

The study involved beneficiaries of the National Health Insurance scheme (covering about 77% of the French population, or 51.5 million people) using the Système National des Données de Santé (French National Health Data System) of individual anonymized pharmacy claims data.^3,4^ The research group has permanent regulatory access to the data from the French National Health Data System (French decree No. 2016-1871 of December 26, 2016, on the processing of personal data called *National Health Data System* and French law articles Art. R. 1461-13 and 14). No informed consent was required because data are anonymized.

The numbers of individuals using aflibercept or ranibizumab were determined by week for the first 23 weeks of 2018, 2019, and 2020. Individuals using bevacizumab were not included; this drug is rarely prescribed and used exclusively in hospitals in France. The study involved the 2 weeks preceding lockdown (March 2 to 15, 2020; weeks 10-11 of the year), 8 weeks of lockdown (March 16 to May 10, 2020; weeks 12-19 of the year), and the first 4 weeks of reopening (May 11 to June 7, 2020; weeks 20-23 of the year). Expected numbers of users per week during these periods of interest were extrapolated from the mean number of users recorded during corresponding weeks in 2018 and 2019. A ratio of 1.16, estimated over weeks 2 to 8 (the mean number of users per week in 2020 divided by the mean number of users per week in 2018 and 2019), was applied to account for the increase in prescriptions between 2018 and 2020. Differences between observed and expected numbers of patients using anti-VEGF were computed each week during periods of interest, overall, and after restrictions to only those using the drugs for the first time (ie, with no reimbursements for IVT anti-VEGF agents during the previous year). All analyses were performed using SAS software, version 9.4 (SAS Institute Inc).

## Results

The study found that 33 292 individuals used IVT anti-VEGF drugs in 2020 before lockdown (20 146 women [60.5%]; mean [SD] age, 77.3 [11.1] years), 87 316 during lockdown (52 461 women [60.1%]; mean [SD] age, 76.8 [11.0] years), and 63 020 during reopening (38 593 women [61.2%]; mean [SD] age, 77.5 [11.0] years) (Table). Compared with expected numbers, observed numbers of individuals using IVT anti-VEGF markedly decreased by up to 47.1% (a decrease of 7432 patients) during the 5 first weeks of lockdown (weeks 12-16, 2020) and remained at a low level until the last week of lockdown (−24.9% [4424 fewer patients] during week 19). During the 8 weeks of lockdown, the shortfall represented a decrease of 46 381 injections. A gradual but incomplete recovery was observed in the first 4 weeks of reopening (difference of −21.9% [4247 fewer patients] in week 20 to −4.2% [723 fewer patients] and −3.5% [581 fewer patients] in weeks 22 and 23). Baseline sex and age characteristics of the patient cohort remained similar for each period (Table; Figure). The decrease was particularly marked (−65.3%) for treatment initiations during lockdown. This decrease corresponds to a total of 8169 fewer treatment initiations during the lockdown period. A gradual recovery was observed during reopening (Table).

**Table.** Time Course of Intravitreal Anti–Vascular Endothelial Growth Factors Reimbursements Between the 2 Weeks Preceding Lockdown Through the First 4 Weeks of Reopening in the Coronavirus Disease 2019 Pandemic

Week No.	Patients in 2020, No.		Difference of observed − expected, No. (%)
	Observed	Expected	
**Period before lockdown**			
Week 10 (March 2-8)	16 114	16 171	−57 (−0.4)
Week 11 (March 9-15)	17 178	17 386	−208 (−1.2)
Total	33 292	33 557	−265 (−0.8)
Female, %	20 146 (60.5)	20 372 (60.7)	NA
Age, mean (SD), y	77.3 (11.1)	77.1 (11.1)	NA
New users, No.^a^	2993	3128	−135 (−4.3)
**Lockdown period**			
Week 12 (March 16-22)	14 978	17 318	−2340 (−13.5)
Week 13 (March 23-29)	10 118	16 751	−6633 (−39.6)
Week 14 (March 30-April 5)	8345	15 777	−7432 (−47.1)
Week 15 (April 6-12)	9099	17 294	−8195 (−47.4)
Week 16 (April 13-19)	9018	16 355	−7337 (−44.9)
Week 17 (April 20-26)	11 199	16 209	−5010 (−30.9)
Week 18 (April 27-May 3)	11 199	16 209	−5010 (−30.9)
Week 19 (May 4-10)	13 360	17 784	−4424 (−24.9)
Total	87 316	133 697	−46 381 (−34.7)
Female, %	52 461 (60.1)	82 069 (61.4)	NA
Age, mean (SD), y	76.8 (11.0)	77.2 (11.1)	NA
New users, No.^a^	4343	12 512	−8169 (−65.3)
**Reopening period**			
Week 20 (May 11-17)	15 188	19 435	−4247 (−21.9)
Week 21 (May 18-24)	15 531	18 174	−2643 (−14.5)
Week 22 (May 25-31)	16 484	17 207	−723 (−4.2)
Week 23 (June 1-7)	15 817	16 398	−581 (−3.5)
Total	63 020	71 214	−8194 (−11.5)
Female, %	38 593 (61.2)	43 828 (61.5)	NA
Age, mean (SD), y	77.5 (11.0)	77.3 (11.1)	NA
New users, No.^a^	4463	4692	−229 (−4.9)

Abbreviations: NA, not applicable.

^a^ New use of intravitreal anti–vascular endothelial growth factors was defined by the absence of any such reimbursement during the previous year.

**Figure.**
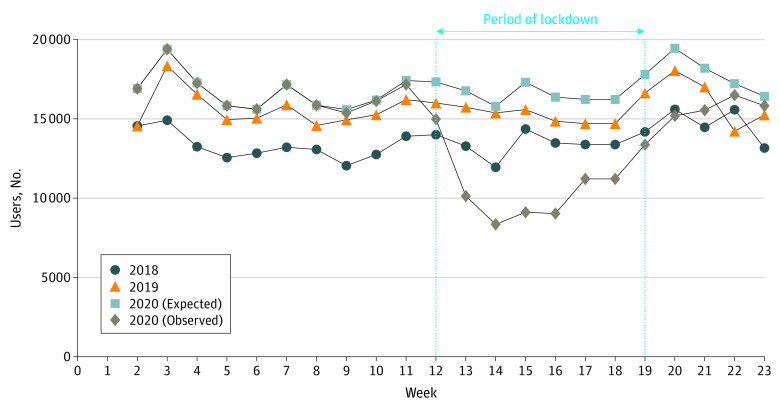
Comparisons of Dispensing by Week and Year Number of patients with at least 1 dispensing of intravitreal anti–vascular endothelial growth factors by week during weeks 2 to 23, 2018, 2019, and 2020, with comparisons of observed and expected numbers (data for National Health Insurance General Scheme beneficiaries, the main French national health insurance scheme, which covers about 77% of the French population).

## Discussion

This study shows a relatively marked decrease in IVT anti-VEGF dispensing during lockdown, which was not completely compensated during the first 4 weeks after unlocking. Extrapolation of these data to the entire French population leads to an estimated decrease of 60 000 injections, including 10 500 initiations of IVT anti-VEGF therapy during the 8 weeks of lockdown. These figures must be interpreted relative to the estimated 85 000 IVT anti-VEGF injections per month in France, including 8000 treatment initiations.^1^ Limitations include the inability to determine whether these findings are associated with any permanent visual acuity loss.

The decrease in IVT anti-VEGF dispensing during the lockdown period could be explained by patients’ difficulty accessing an ophthalmology department or the ophthalmologist’s decision to postpone injections during the pandemic because of difficulties in disease monitoring, which usually include periodic visual acuity and retina examinations, imaging, or both. Most IVT anti-VEGF therapies, particularly with neovascular age-related macular degeneration,^5,6^ should not be delayed, because such delays can lead to permanent visual acuity loss. We believe this situation should continue to be monitored closely, to determine if persistent delays result in longer waiting times to obtain appointments for these treatments. Ideally, the health care system will work on approaches to try to ensure continuity of ophthalmological care in the event of future epidemics.
